# Genome-Wide Identification and Functional Exploration of SBP-Box Gene Family in Black Pepper (*Piper nigrum* L.)

**DOI:** 10.3390/genes12111740

**Published:** 2021-10-29

**Authors:** Jing Li, Rui Fan, Baoduo Wu, Xunzhi Ji, Chaoyun Hao

**Affiliations:** 1 Spice and Beverage Research Institute, Chinese Academy of Tropical Agricultural Sciences, Wanning 571533, China; xys8619@163.com (J.L.); tlfr83@163.com (R.F.); 0204114089@163.com (B.W.); 18876794730@163.com (X.J.); 2 Key Laboratory of Genetic Resources Utilization of Spice and Beverage Crops, Ministry of Agriculture, Wanning 571533, China; 3 Hainan Provincial Key Laboratory of Genetic Improvement and Quality Regulatioin for Tropical Spice and Beverage Crops, Wanning 571533, China; 4 Academician Sim Soonliang of Hainan Province Research Station, Wanning 571533, China

**Keywords:** *Piper nigrum*, SBP-box gene family, co-expression network, miR156

## Abstract

Black pepper (*Piper nigrum* L.), is dubbed “the King of Spices”. However, the lack of genic knowledge has limited the understanding of its physiological processes and hindered the development of its molecular breeding. The SBP-box gene family is an important family in plant development and integrates multiple physiological processes. Here, we made a genome-wide identification of the pepper SBP-box gene family to provide evolutionary and functional information about this conserved transcription factor. In total, 34 *SBP* genes were identified in pepper. All these pepper *SBP* genes were clustered into eight groups, and one pepper group was not found in *Arabidopsis thaliana*. Segment duplications played the most important role in the expansion process of pepper *SBP* genes, and all these duplications were subjected to purifying selection. Half of pepper *SBP* genes were found miR156 target sites, and 17 miR156s were predicted. The tissue expression analysis revealed the differential expression of pepper *SBP* genes. Eleven *SBP* genes were found in four co-expression networks, and the GO enrichment further provides a functional prediction for pepper *SBP* genes. This study lays a foundation for further studies of pepper and provides a valuable reference for functional mining of pepper *SBP* genes.

## 1. Introduction

Transcription factors (TFs), which are proteins that regulate gene expression by activating or repressing the transcription of downstream target genes, play essential roles in the regulatory networks of many development processes in all living organisms [1,2]. Different families or subfamilies of TFs were found in plants according to their structure of DNA-binding domain, such as WRKY, MYB, MADS-box, DOF, and NAC transcription factor families. SQUAMOSA promoter-binding protein (briefly: SBP-box) or SBP-like (SPL) genes encode a type of TF family that is conserved in green plants. *SBP* genes were first found in *Antirrhonum majus* to regulate the expression of some MADS-box genes that play a critical role in floral development [3]. Since then, studies continue to reveal that *SBP* genes play critical roles in the regulation of plant development processes, signal transduction, defense processes from monocyte algae to flowering plants, and especially the outstanding roles in angiosperms flowering development and time-dependent phase transformation processes [4,5,6,7]. The SBP-box gene family play important roles in plant growth and development of the regulation network, and it is a key link to explore the molecular mechanism of plant growth and development [8]. In addition, the evolutionary patterns of the SBP-box gene family are highly consistent with the evolution of angiosperms, so it is also an important resource to reveal the plant lineage-specific evolution process.

The SBP domain, which contains about 75 amino acid residues (aa), is the typical structure for all SBP TFs. As studies revealed, the SBP domain is sufficient to bind to the GTAC core motif [4,5,6,7]. Generally, the SBP domain contains three characteristic structures: two zinc fingers and a nuclear localization signal (NLS). Mostly, the NLS overlaps with the second zinc finger [9]. In addition, some *SBP* genes are targets of miR156. The miR156 mature sequences can reduce the protein expression level at the transcriptional or translational stage by complementarily binding to their target mRNAs [10,11]. To angiosperms, a considerable part of *SBP* genes can be regulated by miR156s. It has been identified that 10 of 16 *AtSPL* genes are potential targets of miR156/157 (collectively known as miR156). Some *SBP* genes regulated by miR156s are involved in time-dependent or tissue-specific development processes. For example, the miR156 and SBP module play critical roles in the juvenile-to-adult phase transition of *A. thaliana* [12,13,14]. In *A. thaliana*, AtSPL3/4/5, which are direct upstream activators of LEAFY, FRUITFUL, and APETALA1, redundantly promote flowering [12]. They also integrate photoperiodic signals and developmental aging in the flowering locus T (FT)/flowering locus D (FD) module process [13]. AtSPL9/15 are regarded as regulators of plastochron and branching [15,16]. AtSPL7 is a regulator of copper homeostasis and responds to light and copper [17]. 

Pepper is a member of the family Piperaceae and is originally native to India. Pepper is known for its medicinal properties and unique aroma and flavor [18]. It is one of the most popular spices, thus also called “the King of Spices”. Different types of pepper products have been widely used in the medical, food, and cosmetics industries [19,20,21]. Due to the huge commercial value and increasing market demand, pepper has become increasingly attractive. Additionally, pepper is an early branch of angiosperms [22], and is likely a key link to explain the formation and expansion of angiosperms. The limited genic studies of pepper led to the lack of knowledge about the physiological processes such as growth and development, and stress response. At the same time, it also limits the development of worldwide pepper molecular breeding. To improve the yield, quality, and disease resistance, it is extremely necessary to perform more research on genes playing critical roles in multiple important physiological processes.

*SBP* genes have been identified in many species through the bioinformatic method, however, there has been no relevant reports in pepper. Fortunately, the high-precision pepper genome assembly allows more in-depth research to be conducted on the pepper SBP-box gene family [23]. In this study, we performed a genome-wide investigation of the SBP-box gene family in pepper. HMM and BLASTP searches were both adopted to predict *SBP* genes in a genome-wide way in pepper; the phylogenetic tree was reconstructed by both ML (maximum likelihood) and NJ (neighbor-joining) methods; motif analysis, and multiple sequence alignment were used to parse the protein structure; the duplications of pepper *SBP* genes were predicted and their selection pressure was calculated; miR156s were identified and the corresponding miR156 binding sites were predicted in pepper *SBP* genes; tissue expression pattern of pepper *SBP* genes was displayed; and, at last, functional analysis including construction of a co-expression network and a GO enrichment were carried out. Based on the above analysis, both evolutionary and functional information of the pepper SBP-box gene family was revealed, which is expected to be useful for more in-depth studies in the future.

## 2. Methods

### 2.1. Data Sources

The genomic and proteomic sequences of pepper were obtained from the assembly of our laboratory and have been deposited at the website (http://cotton.hzau.edu.cn/EN/download.php accessed on 30 February 2021). The *A. thaliana* genomic and proteomic sequences were obtained from TAIR database (accessed on 30 March 2021) [24]. The gene expression data for pepper was from previous experiment data of our laboratory and has been deposited in the SRA database under BioProject number PRJNA529760 (30 February 2021). The expression data of *A. thaliana* were obtained from TAIR database (30 March 2021).

### 2.2. Identification and Characterization of Pepper SBP Genes

Both HMM and BLASTP searches were performed to accurately predict the pepper *SBP* genes [25,26]. For HMM search, the SBP-specific HMM profile (PF03110) was obtained from the Pfam database [27], and the HMMER toolkit was adopted to find suspected *SBP* genes. In addition, the well-characterized *A. thaliana* SBP protein sequences were collected from PlantTFDB as queries for BLASTP searches (*e*-value ≤ 1 × 10^−10^). The SBP structural integrity was confirmed using the Simple Modular Architecture Research Tool (SMART) [28]. The online toolkit ExPASy Proteomics Server was used to predict the physicochemical properties [29], including protein length, molecular weight (MW), and isoelectric point (pI).

### 2.3. Phylogenetic Analysis and Conserved Motif Prediction

Multiple sequence alignment of SBP protein sequences was performed using Multiple Sequence Comparison by Log-Expectation (MUSCLE) (v3.8) and ClustalX (v2.0) [30,31]. A neighbor-joining (NJ) tree was generated using MEGA 11 with 1000 bootstrap replicates [32]. To make a more reliable phylogenetic analysis, IQTREE was used to reconstruct the maximum likelihood (ML) tree [33]. The final phylogenetic relationship was based on the results obtained from the two methods, and the visualization was conducted by EvolView software [34]. The motifs of pepper SBP proteins were predicted using an online toolkit of Multiple Expectation Maximization for Motif Elucidation (MEME) [35]. All whole long SBP proteins were adopted as queries, and the parameters were set as follows: minimum width was 6, maximum width was 150, motif number was 10, and the minimum number of sites was 2.

### 2.4. Chromosomal Localization and Detection of Gene Duplication

According to the position on chromosomes, the location of each *SBP* gene was visualized. Both tandem duplications and segmental duplications here were predicted according to the method described in the Plant Genome Duplication Database [36]. MCScanX was used to detect the segment duplicate alignments [37], and the all-against-all BLASTP comparison (*e*-value ≤1 × 10^−10^) was performed to supply the input data for MCScanX analysis. The segment duplications were manually confirmed from alignments produced from MCScanX analysis. Tandem duplications were accepted as those genes next to each other or separated by one unrelated gene.

### 2.5. Estimation of Synonymous (Ks) and Nonsynonymous (Ka) Substitutions per Site and Their Ratio (Ka/Ks)

The *SBP* genes that satisfied segment duplications were used to estimate *Ka* (the number of nonsynonymous substitutions per nonsynonymous site), *Ks* (the number of synonymous substitutions per synonymous site), and their ratio (*Ka/Ks*). Coding sequences from segmentally duplicated *SBP* genes were aligned using PRANK [38]. The estimation of *Ka*, *Ks,* and *Ka/Ks* were developed using KaKs_Calculator (v2.0) [39], and the MA (a model that averaged the result from many classical calculation models provided by KaKs_Calculator) model was adopted. The *Ka/Ks* value can reflect the selective pressure of duplicated gene pairs, and the *Ks* value can reflect the divergence time for duplication events.

### 2.6. MicroRNA and Target Prediction

The miR156/157 sequences were downloaded from the miRBase database [40]. Due to the high similarity of miR156 and miR157, they were collectively referred to as miR156 family. The prediction of pepper miR156 was performed according to the sequence similarity. The precursor sequences of *miR156* genes were used as queries to blastn against the pepper genome database, and the sequences were further confirmed using MFold to make sure a suitable RNA secondary structure with relative higher negative minimal free folding energy [41,42]. The psRNATarget program was used to predict target sequences in pepper *SBP* genes, and the mature miR156 sequences downloaded from miRBase were used as queries [43].

### 2.7. Tissue Expression, WGCNA Analysis, GO Enrichment, and Co-Expression Network Construction

The expression data of pepper *SBP* genes in root, stem, leaf, flower, and fruit of four periods were used to construct tissue expression profile. The R package WGCNA was used to find the correlations between *SBP* genes and other genes [44]. BLASTP was made against the Uniport dataset, and DAVID was used to make GO enrichment analysis [45]. The co-expression network was constructed using Cytoscape [46].

## 3. Results

A genome-wide analysis result in the identification of 34 *SBP* genes, named *PnSBP1* to *PnSBP34* (Table 1), in the pepper genome. All identified *SBP* genes have been further confirmed using SMART to make sure complete SBP domain structures were obtained. Furthermore, some basic protein parameters including protein length, isoelectric point, and molecular weight were analyzed through the calculating of ExPASy. The protein length was ranging from 177aa to 1101aa, the molecular weight was from 19.80kDa to 120.90kDa, and the isoelectric point was from 6.30 to 10.28 (Table 1).

### 3.1. Phylogenetic Analysis

**Figure 1:**
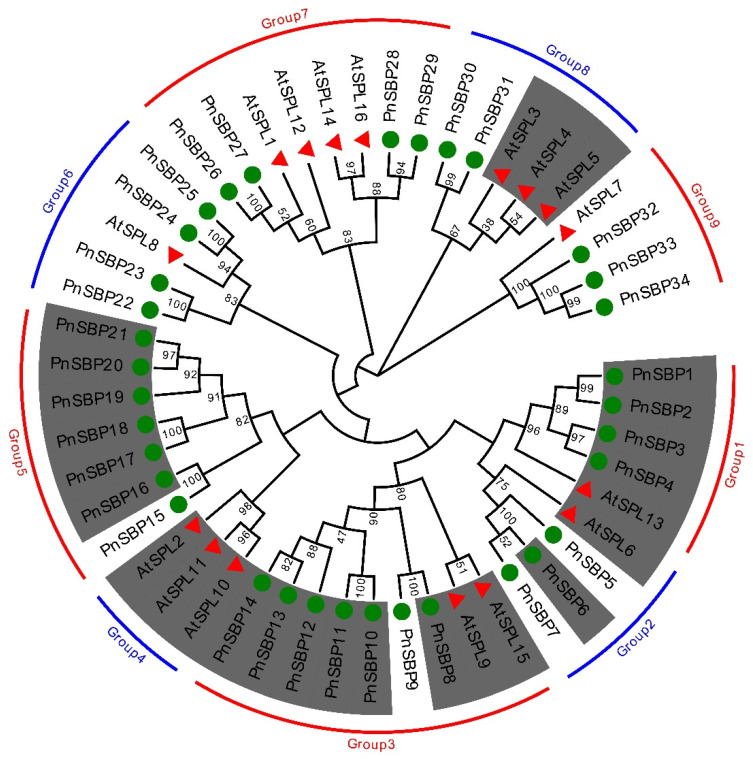
The phylogenetic tree of pepper *SBP* genes. The red triangles refer to *SBP* genes of *A. thaliana*; the green balls refer to pepper *SBP* genes. Each group is highlighted with a color stripe. The *SBP* genes with miR156 target site were marked with dark background color.

To better understand the evolutionary trajectory of pepper *SBP* genes, a phylogenetic analysis of 34 pepper SBPs plus 16 *A. thaliana* SPLs was implemented (Figure 1). All these *SBP* genes were divided into nine groups according to the phylogenetic analysis, namely, group 1 to group 9. The bootstrap values were most concentrated from 80 to 100, which reflects a reliable phylogenetic relationship. Most groups were able to find both *A. thaliana* and pepper homologous genes, while group 4 was unique in *A. thaliana* and group 5 was specific to pepper. In addition, except group 8, all pepper groups have gene copies more than or equal to *A. thaliana*.

### 3.2. Multiple Sequence Alignment and Conserved Motif Analysis

**Figure 2:**
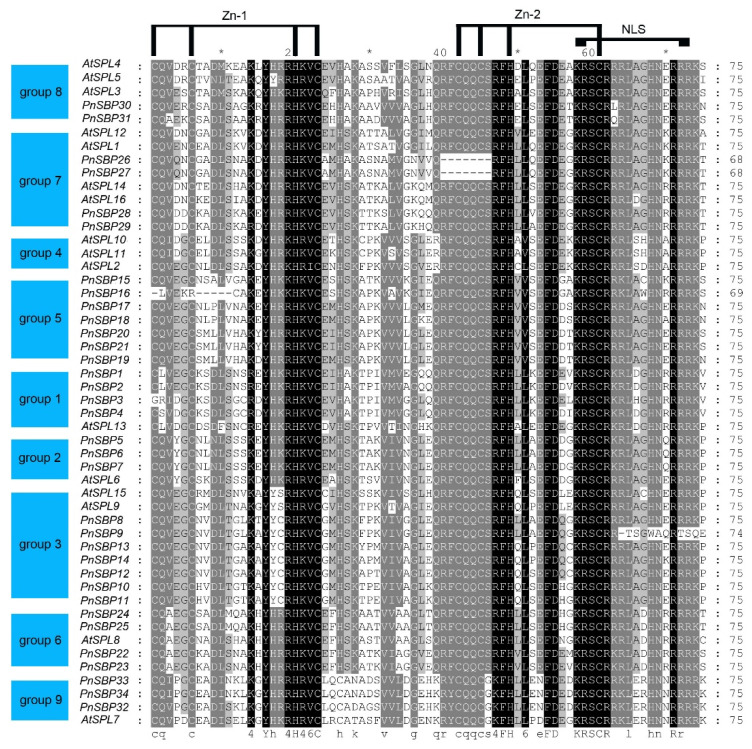
The multiple alignment of SBP-specific domain. Zn-1, Zn-2, and NLS are highlighted on the top.

A multiple sequence alignment was also conducted for the SBP domain structures of the pepper and *A. thaliana SBP* genes (Figure 2). Two Zn-fingers and one NLS were highly conserved in pepper SBP proteins, even so, we still retained five highly suspected sequences (PnSBP26, PnSBP27, PnSBP16, PnSBP3, PnSBP9) with defects in the three typical structures. The typical structure of the two Zn-fingers was Cys-Cys-His-Cys, and the second Zn-finger overlapped with the NLS. In addition, the sequences in the same group were more similar, and they often share group-specific nucleic acids.

**Figure 3:**
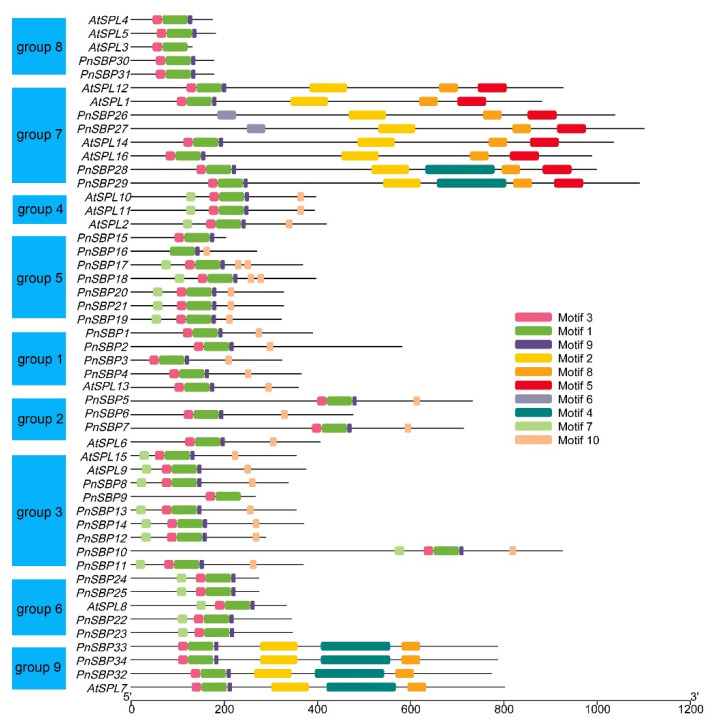
The conserved motifs in pepper *SBP* genes. The genes in the same group were drawn together, and the group number was marked to the left.

We further predicted conserved motifs for all SBP proteins (Figure 3). A total of 10 motifs were found. Three motifs constitute the structure of SBP-specific domain (motif 1/3/9). The motif number was consistent with protein length; the relatively longer SBP proteins in group 7/9 were rich in motifs, sharply contrasting with the shorter SBP proteins in group 8. Some motifs were conserved across groups, such as motif 7/10; some motifs were group-specific, such as motif 5/6 were unique to group 7.

### 3.3. Chromosomal Localization and Gene Duplication Analysis

**Figure 4:**
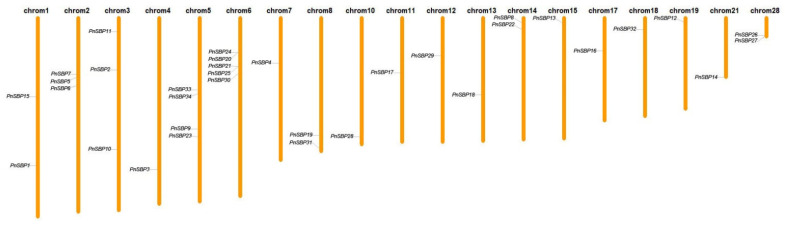
The gene distribution in the pepper genome.

The chromosomal distribution of 34 *SBP* genes throughout the pepper genome was plotted. All *SBP* genes were spread unequally on 19 chromosomes (Figure 4). The gene duplications were recorded in a table (Table S1). After the MCScanX searching and manually screening, 28 pairs of duplicated *SBP* genes were found in accord with segment duplications. Most segment duplications were found between different chromosomes, while a smaller number of segment duplications were also found within the same chromosome. Compared to segment duplication, the tandem duplication had little effect on *SBP* gene expansion in pepper. Only two pairs of tandem duplications were discovered (*PnSBP24* and *PnSBP20*; *PnSBP25* and *PnSBP21*).

**Figure 5:**
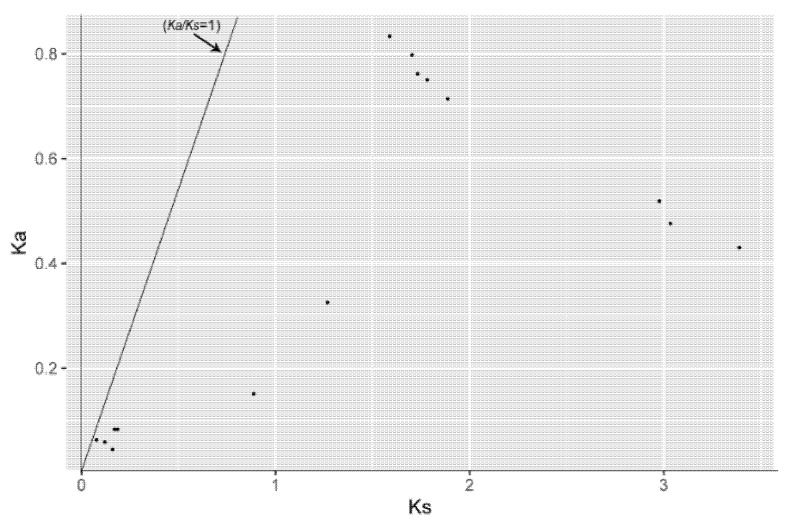
The *Ka*, *Ks* value of duplication gene pairs, and the *Ka/Ks* = 1 line was plotted in the graph.

The results of segment duplications were highly consistent with the phylogenetic grouping scheme that each pair of segmentally duplicated genes belong to the same evolutionary group. To further explore the evolutionary constraints on the pepper *SBP* genes, nonsynonymous substitution rate (*Ka*), synonymous substitution rate (*Ks*), and their ratio (*Ka/Ks*) were calculated for the segment duplication gene pairs. According to the *Ka/Ks* result (Figure 5), all *SBP* genes follow purifying selection (*Ka/Ks* < 1) which reflects they tend to rule out harmful mutations. 

### 3.4. Prediction of MiR156 and Their Target Sites

**Figure 6:**
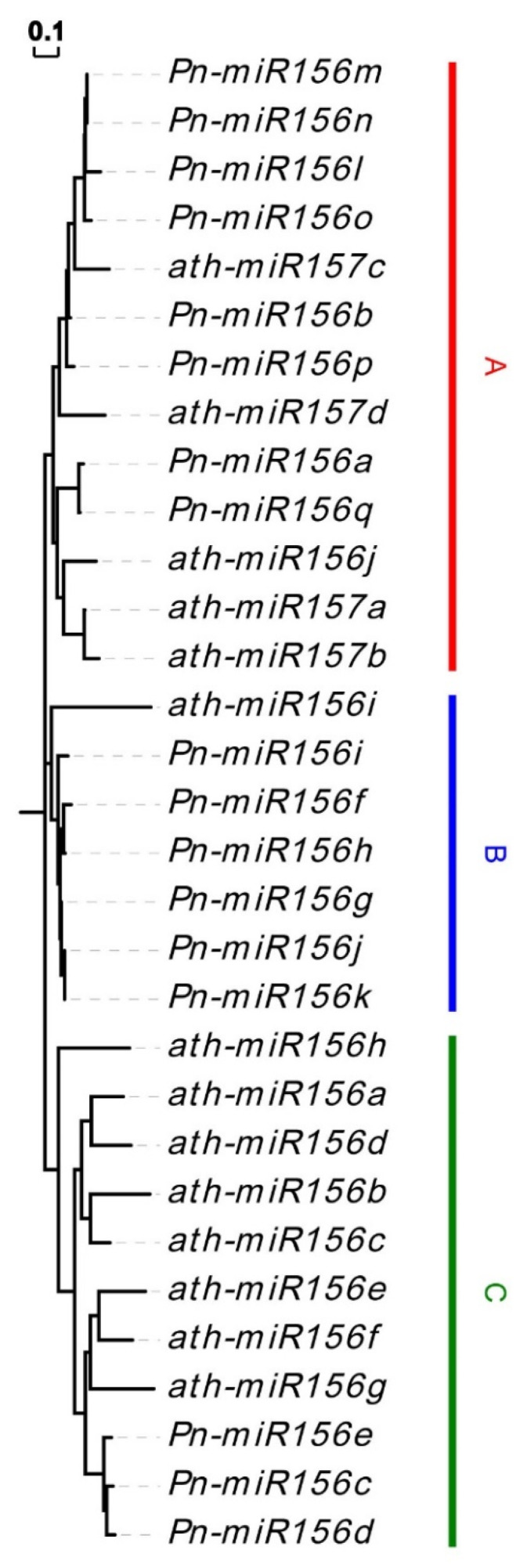
The phylogenetic tree of miR156s, three groups are highlighted in different color stripes. The *A. thaliana* sequences start with ath- and the pepper sequences start with Pn-. Three phylogenetic groups were marked by (**A**–**C**).

The regulation of miR156 is a significant characteristic for *SBP* genes, and the miR156 sequences of many species from experimental identification or in silico prediction have been deposited on the miRBase database. Based on the similarity among homologous sequences, we performed a genome-wide search of miR156 precursor sequences in pepper. To further confirm the accuracy of the results, we predicted the RNA secondary structure for all predicted miR156 precursor sequences (Figure S1). Through the above analysis, 17 miR156s (*Pn-miR156a* to *Pn-miR156q*) were identified (Table S2). A phylogenetic analysis was also conducted on pepper miR156s. According to the phylogenetic tree, three groups were obtained from 17 Pn-miR156s (Figure 6), namely groups A to C. There are eight Pn-miR156s in group A, six in group B, and three in group C. The targets of miR156s were also predicted in pepper *SBP* genes. Seventeen pepper *SBP* genes were found in miR156 binding sequences (Table S3), leading to a 1:1 ratio of with and without miR156 target sites of pepper *SBP* genes. In general, the distribution of target sites in pepper and *A. thaliana* is similar, and the *A. thaliana* group with target site can also find targets in its pepper homologous group. However, the biggest difference was in group 8, where the target locus was present in *A. thaliana* but not in pepper. We do not exclude the possibility that the present genome assembly version may be inaccurate in predicting the target loci of 3′ UTR regions (former studies have revealed that the target loci of homologous genes in group 8 are located in 3′ UTR, while the target sites of other groups are generally located in CDS [6]). The pepper sequences with miR156 target sites were marked with background color in Figure 1. There are four groups in which miR156 target sites were found. Some sequences in group 2 and group 3 had lost target sites.

### 3.5. Tissue Expression and WGCNA Analysis

**Figure 7:**
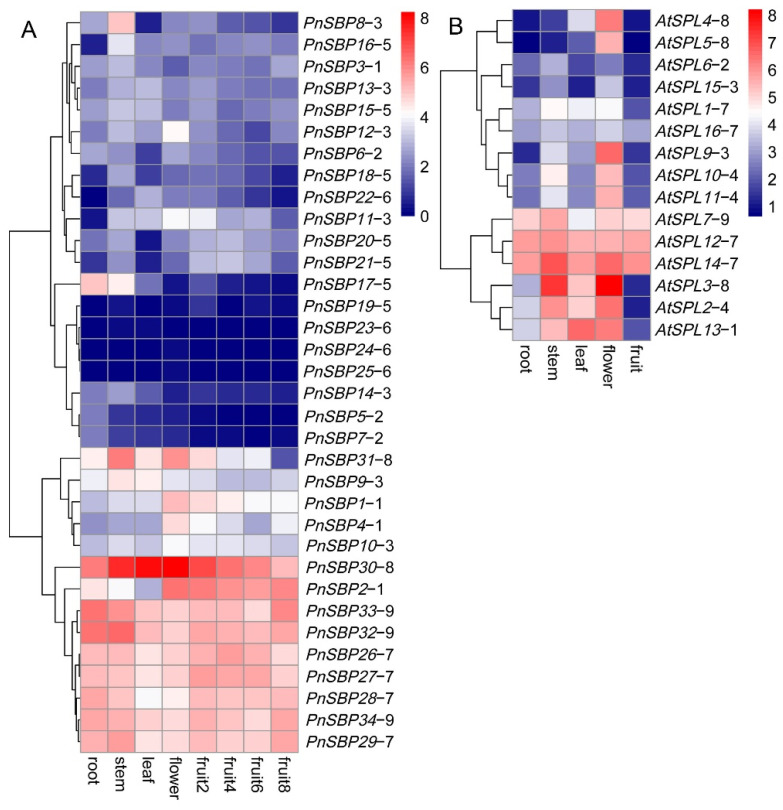
The heatmap of tissue expression of pepper *SBP* genes. The FPKM values have been log2 transformed. The group number of each gene was marked after the gene name. (**A**) The expression patterns of pepper *SBP* genes. (**B**) The expression patterns of *A. thaliana* genes.

**Figure 8:**
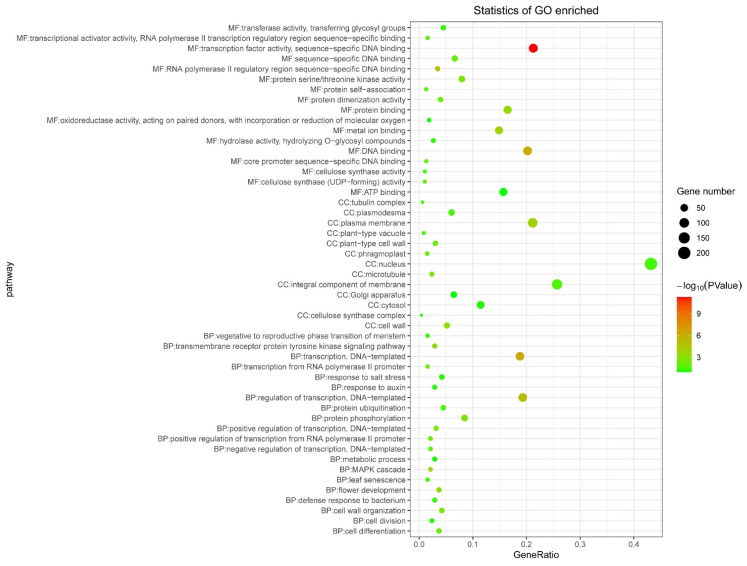
The bubble diagram of the top related genes in pepper *SBP* gene co-expression network. The GeneRatio refers to the ratio of genes enriched to all genes in a same pathway.

**Figure 9:**
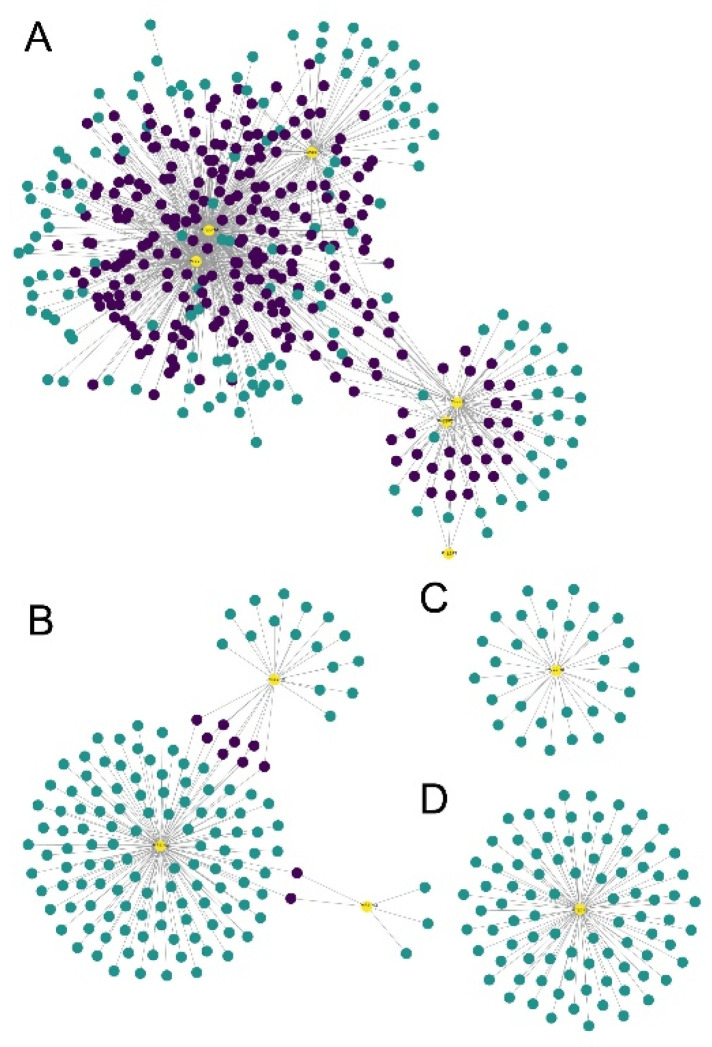
The co-expression networks of pepper *SBP* genes. Four networks were obtained and shown in (**A**–**D**).

We extracted all the edges containing *SBP* genes from the network generated by WGCNA and then screened out the edges with the top 1000 correlation values (Figure 7). The 1000 edges contained 15 *SBP* genes and 684 associated genes. According to the expression values, a cluster analysis was conducted on the 699 genes (15 *SBP* genes plus 684 associated genes). Further, we made a GO enrichment analysis among the 699 genes. The bubble diagram below showed the top 20 enriched GO terms (Figure 8). In general, a lot of transcription factors related terms were obtained, and some terms related to flower development and stress responses were also obtained in BP (Biology Process) terms. To further reveal the functional relationships among the 699 genes, a co-expression network analysis was made. As shown in the graph (Figure 9), four networks (A, B, C, D) with a relatively larger number of associated genes were obtained. In the 4 networks, 11 SBPs were dispersed alone or connected with several other *SBP* genes. Network A had the largest number of associated genes, with up to 6 *SBP* genes connected by multiple intermediate genes (the genes that connect more than one *SBP* gene). Among the 6 *SBP* genes in network A, one gene in group 1, two genes in group 2, two genes in group 3, and one gene in group 5. The other 3 networks were relatively smaller, and networks C and D found no connections with other *SBP* genes.

## 4. Discussion

Pepper, as a natural spice crop, is in great demand not only in the food field, but also in the cosmetics and health industry [47,48]. However, genic mining for pepper is still limited, and more fundamental research is needed to provide data support in the pursuit of high yield, high disease resistance, high quality, and other excellent agronomic characters. The SBP-box gene family is important in regulating flower development, vegetative to reproductive phase transition, leaf development, plastochron, tillering/branching, panicle/tassel architecture, fruit ripening, fertility, signal transduction, and defense processes [4,5,6,7,12,13,14,15,16,17]. Particularly, *SBP* genes usually form an important regulatory module with miR156, which plays a hub-like role in plant growth and development of the regulatory network [8]. The module of SBP/miR156 integrates many endogenous and environmental cues, such as sugars, higher ambient temperature, some abiotic stresses, gibberellic acid, floral inductive photoperiod, and some hormones such as auxin and light to serve as a regulatory hub to determine the balance of two goals: to complete reproduction, or to grow more branches/leaves [8]. At present, little genic research has been done on pepper, and the knowledge about pepper physiological processes is very limited. Therefore, to improve crop agronomic traits and promote the development of molecular breeding, it is necessary to study the SBP-box gene family in pepper.

There are 34 *SBP* genes identified in pepper, twice as many as *A. thaliana*. Recent whole-genome duplication events are likely responsible for a large number of *SBP* genes in pepper [23]. This study did not find any segment duplications from earlier whole-genome duplication events, which is consistent with previous findings [23]. The pepper SBP-box gene family was amplified in a large scale after the recent duplication event, which resulted in genetic redundancy in some groups. Genes were doubled after whole-genome duplication, and gene redundancy led to new functionalization, sub-functionalization, and loss of function [49,50]. The tissue expression profile exhibited most of the pepper *SBP* genes which are probably functional virtually, which means that the regulation network of *SBP* genes in pepper is relatively complex. Compared with *A. thaliana*, some groups have a significant expansion in pepper; for example, group 1 and 6 both have only one copy in *A. thaliana*, but there are four copies in pepper; group 2 and group 9 both have one copy in *A. thaliana*, but there are three in pepper; group 3 has two copies in *A. thaliana*, but there are seven copies in pepper. More copies make the regulatory network of redundancy copies more complicated. For group 9, the three copies display similar expression profiles and they are all highly expressed with low tissue differences. Groups 1, 2, and 3 have both high expression copies and low ones. For group 6, two of its three members had no expression detected. These results indicate that sub-functionalization, neo-functionalization, or non-functionalization may have occurred among the duplicated copies, leading to functional differentiation among the redundant members. In addition, there may be significant functional differences between pepper and *A. thaliana SBP* genes. Group 4, which plays critical roles in *A. thaliana*, has no homologous gene in pepper. Conversely, group 5, which is missing in *A. thaliana*, has as many as seven copies in pepper, five copies of which have detected tissue differential expression patterns. Is the function of missing group 4 in pepper compensated by other genes? Have the genes in group 5 obtained new functions in pepper? Solving these problems is very interesting and necessary.

The regulation of miR156 is also a significant feature for pepper *SBP* genes, and the study of these genes may help to solve some long-standing problems in the pepper industry. Because *SBP* genes were found both with and without miR156 binding sites, it is probable that some genes have lost their target sites. Like other angiosperms [4,5,6], pepper has several groups with relatively longer protein lengths, no miR156 binding sites, and house-keeping-like expression patterns. These *SBP* genes are highly conserved in angiosperms and probably functionally conserved in land plants. Previous studies have shown that the *SBP* genes regulated by miR156 are often tissue-specific [6], which is in high accordance with our study. *AtSPL7* is a gene confirmed to this form, and has been identified as a regulator of copper homeostasis and responses to light and copper [17]. Three *AtSPL7* homologous genes were found in pepper, and the tissue expression patterns suggest that they may have similar functions to *AtSPL7*. In addition, *SBP* genes may be important regulators in pepper development. The genes in group 8 (*PnSBP30* and *PnSBP31*) are highly expressed in many tissues; their homologous genes in *A. thaliana* are key regulators in flower development and time-dependent pathways [13,14], Pepper is considered to be a plant with flowering degeneration, and there are few studies on its flowering regulation mechanism. It is hoped that pepper can be harvested with the characteristics of consistent flowering, and flowering regulation genes may be the key to solving these problems. *AtSPL8* (the homologous gene of *PnSBP22*) has been reported to be involved in gynoecium and another development, male fertility, and GA-mediated development processes [51,52]. Thus, it can be seen, pepper *SBP* genes are likely functional like other angiosperms, and may be critical regulators in pepper growth and development.

According to previous studies, the *A. thaliana SBP* genes have evolved functional diversity and formed a complex regulatory network [53,54]. The pepper *SBP* genes also displayed obvious tissue differential expression patterns. This reflects that pepper *SBP* genes may play roles in a variety of developmental processes and physiological pathways. The pepper *SBP* genes have been functionally differentiated, 11 *SBP* genes clustered into four co-expression networks and no connection was found among the four networks. Since the co-expression networks of multiple *SBP* genes in Figure 9A are highly intersected and aggregated into a very large co-expression network, we speculate that these *SBP* genes may have functional correlations.

The study of pepper is of great significance to the evolution of angiosperms. Pepper has a special place in the evolutionary history of plants. According to APG IV, Piperaceae has a phylogenetic taxonomic position independent of dicotyledons and monocotyledons and belongs to a branch near the basal angiosperms. In contrast to the highly evolved floral organs of many angiosperms, Piperaceae is thought to be vestigial [23]. Angiosperms probably emerged in the early Cretaceous and expanded explosively, evolving into multiple taxa and diverse phenotypes. Former studies had revealed that the SBP-box gene family is a highly conserved gene family in plants, and its expansion pattern is highly consistent with the evolutionary process of angiosperms and is an important genic driving force for angiosperm evolution [6]. This study found that the SBP-box gene family of pepper is highly differentiated and results in a grouping scheme similar to that of other angiosperms. This result supports that the major branches of the SBP-box gene family have emerged and been preserved in the early angiosperms, similar to the previous study [6]. After the formation of angiosperms, the SBP-box gene family expanded in a lineage-specific manner. The pepper SBP-box gene family has undergone significant expansion, and specific groups, higher copy number, and a more complex regulatory mechanism of intra-group members have emerged compared with *A. thaliana*. These may be important genic drivers to promote the formation of unique physiological characteristics and competitive advantages of pepper. There are still many questions about the pepper SBP-box gene family to be answered, and further exploration is not only beneficial to improve the understanding of pepper, but also a key step to explore the evolution of angiosperms.

## 5. Conclusions

Our results provide a comprehensive phylogenetic classification of the pepper SBP-box gene family. Thirty-four *SBP* genes were identified in the pepper genome, and the phylogenetic analysis revealed that they can be classified into eight groups. The phylogenetic difference between *A. thaliana* and pepper was relatively significant, in that one *A. thaliana* group was missing in pepper and one group was pepper specific. Compared with *A. thaliana*, significant gene expansion was found in some groups, resulting in a large number of redundant genes in these groups. The distribution of genes regulated by miR156 in pepper was similar to that in *A. thaliana*, but some genes lost regulatory sites. The genes regulated by miR156 displayed significant tissue differential expression patterns, and a large number of redundancies also made the regulatory network of the pepper SBP-box gene family more complex. The specific gene expansion and the redundancies functional changes provided the genic impetus for the evolution of pepper-specific physiological characteristics and competitive advantages. The present findings provide an improved understanding of the evolution and functions of the SBP-box gene family in pepper, and present a framework for further detailed analysis of this gene family.

## Figures and Tables

**Table 1:** The basic information of pepper *SBP* genes.

Names	Gene	Length (aa)	MV (kDa)	pI
*PnSBP8*	*Pn14.164*	337	36.7	8.72
*PnSBP13*	*Pn15.136*	354	38.2	8.62
*PnSBP4*	*Pn7.344*	365	40.3	8.72
*PnSBP28*	*Pn10.2075*	999	110.2	7.29
*PnSBP34*	*Pn5.1721*	787	88.1	6.40
*PnSBP33*	*Pn5.1651*	787	88.0	6.47
*PnSBP32*	*Pn18.304*	774	86.1	6.65
*PnSBP16*	*Pn17.1041*	269	30.7	9.61
*PnSBP9*	*Pn5.2134*	266	30.1	8.49
*PnSBP26*	*Pn28.549*	1038	114.4	6.30
*PnSBP27*	*Pn28.583*	1101	120.9	6.80
*PnSBP29*	*Pn12.1036*	1091	120.6	7.91
*PnSBP15*	*Pn1.1351*	203	23.2	10.28
*PnSBP1*	*Pn1.2504*	389	44.3	8.05
*PnSBP17*	*Pn11.1178*	368	40.0	8.44
*PnSBP18*	*Pn13.776*	396	43.8	9.62
*PnSBP22*	*Pn14.389*	344	38.7	7.63
*PnSBP12*	*Pn19.85*	289	30.9	8.94
*PnSBP5*	*Pn2.1033*	732	80.1	7.82
*PnSBP6*	*Pn2.1339*	476	52.3	8.73
*PnSBP7*	*Pn2.939*	713	78.9	8.66
*PnSBP14*	*Pn21.1417*	370	39.7	7.76
*PnSBP2*	*Pn3.1618*	581	64.4	8.54
*PnSBP10*	*Pn3.4074*	926	102.0	6.38
*PnSBP11*	*Pn3.426*	369	39.7	9.42
*PnSBP3*	*Pn4.2537*	323	35.9	8.43
*PnSBP23*	*Pn5.2416*	346	38.7	7.97
*PnSBP30*	*Pn6.1128*	177	19.8	9.47
*PnSBP24*	*Pn6.473*	274	29.8	8.75
*PnSBP20*	*Pn6.475*	327	35.5	8.43
*PnSBP21*	*Pn6.937*	327	35.6	8.37
*PnSBP25*	*Pn6.939*	274	29.8	8.75
*PnSBP19*	*Pn8.2451*	322	35.2	9.25
*PnSBP31*	*Pn8.2831*	177	19.9	9.18

## Data Availability

Data used in this study are presented in the supplementary information.

## Supplementary Materials

The following are available online at https://www.mdpi.com/article/10.3390/genes12111740/s1, Figure S1: The prediction of RNA secondary structure of pepper miR156s, Table S1: The duplications of pepper *SBP* genes, Table S2: The information of predicted *miR156* genes in pepper genome, Table S3: The distribution of miR156 binding sites in pepper *SBP* genes.

## References

[1] Van Lijsebettens M., Grasser K.D. (2014). Transcript elongation factors: Shaping transcriptomes after transcript initiation. Trends Plant Sci..

[2] Yamasaki K., Kigawa T., Seki M., Shinozaki K., Yokoyama S. (2012). DNA-binding domains of plant-specific transcription factors: Structure, function, and evolution. Trends Plant Sci..

[3] Klein J., Saedler H., Huijser P. (1996). A new family of DNA binding proteins includes putative transcriptional regulators of the *Antirrhinum majus* floral meristem identity gene SQUAMOSA. Mol. Gen. Genet..

[4] Riese M., Höhmann S., Saedler H., Münster T., Huijser P. (2007). Comparative analysis of the SBP-box gene families in *P. patens* and seed plants. Gene.

[5] Yang Z., Wang X., Gu S., Hu Z., Xu H., Xu C. (2008). Comparative study of SBP-box gene family in *Arabidopsis* and rice. Gene.

[6] Li J., Gao X., Zhang X., Liu C. (2020). Dynamic expansion and functional evolutionary profiles of plant conservative gene family SBP-box in twenty two flowering plants and the origin of miR156. Biomolecules.

[7] Zhang H.X., Jin J.H., He Y.M., Lu B.Y., Li D.W., Chai W.G., Khan A., Gong Z.H. (2016). Genome-wide identification and analysis of the SBP-box family genes under *Phytophthora capsici* stress in pepper (*Capsicum annuum* L.). Front. Plant Sci..

[8] Wang H., Wang H. (2015). The miR156/SPL module, a regulatory hub and versatile toolbox, gears up crops for enhanced agronomic traits. Mol. Plant.

[9] Birkenbihl R.P., Jach G., Saedler H., Huijser P. (2005). Functional dissection of the plant-specific SBP-domain: Overlap of the DNA-binding and nuclear localization domains. J. Mol. Biol..

[10] Rogers K., Chen X. (2013). Biogenesis, turnover, and mode of action of plant microRNAs. Plant Cell.

[11] Voinnet O. (2009). Origin, biogenesis, and activity of plant microRNAs. Cell.

[12] Yamaguchi A., Wu M.F., Yang L., Wu G., Poethig R.S., Wagner D. (2009). The microRNA-regulated SBP-box transcription factor SPL3 is a direct upstream activator of leafy, FRUITFULL, and APETALA1. Dev. Cell.

[13] Jung J.H., Lee H.J., Ryu J.Y., Park C.M. (2016). SPL3/4/5 integrate developmental aging and photoperiodic signals into the FT-FD module in Arabidopsis flowering. Mol. Plant.

[14] Cardon G.H., Höhmann S., Nettesheim K., Saedler H., Huijser P. (1997). Functional analysis of the *Arabidopsis thaliana* SBP-box gene *SPL3*: A novel gene involved in the floral transition. Plant J..

[15] Shikata M., Koyama T., Mitsuda N., Ohme-Takagi M. (2009). *Arabidopsis* SBP-box genes *SPL10*, *SPL11* and *SPL2* control morphological change in association with shoot maturation in the reproductive phase. Plant Cell Physiol..

[16] Schwarz S., Grande A.V., Bujdoso N., Saedler H., Huijser P. (2008). The microRNA regulated SBP-box genes *SPL9* and *SPL15* control shoot maturation in *Arabidopsis*. Plant Mol. Biol.

[17] Zhang H., Zhao X., Li J., Cai H., Deng X.W., Li L. (2014). MicroRNA408 is critical for the *HY5*-*SPL7* gene network that mediates the coordinated response to light and copper. Plant Cell.

[18] Salehi B., Zakaria Z.A., Gyawali R., Ibrahim S.A., Rajkovic J., Shinwari Z.K., Khan T., Sharifi-Rad J., Ozleyen A., Turkdonmez E. (2019). Piper species: A comprehensive review on their phytochemistry, biological activities and applications. Molecules.

[19] Takooree H., Aumeeruddy M.Z., Rengasamy K.R.R., Venugopala K.N., Jeewon R., Zengin G.A.-O., Mahomoodally M.F. (2019). A systematic review on black pepper (*Piper nigrum* L.): From folk uses to pharmacological applications. Crit. Rev. Food Sci. Nutr..

[20] Butt M.S., Pasha I., Sultan M.T., Randhawa M.A., Saeed F., Ahmed W. (2013). Black pepper and health claims: A comprehensive treatise. Crit Rev. Food Sci. Nutr..

[21] Dosoky N.S., Satyal P., Barata L.M., da Silva J.K.R., Setzer W.N. (2019). Volatiles of black pepper fruits (*Piper nigrum* L.). Molecules.

[22] Group T.A.P., Chase M.W., Christenhusz M.J.M., Fay M.F., Byng J.W., Judd W.S., Soltis D.E., Mabberley D.J., Sennikov A.N., Soltis P.S. (2016). An update of the angiosperm phylogeny group classification for the orders and families of flowering plants: Apg IV. Bot. J. Linn. Soc..

[23] Hu L., Xu Z.A.-O., Wang M.A.-O., Fan R., Yuan D.A.-O., Wu B., Wu H., Qin X., Yan L., Tan L. (2019). The chromosome-scale reference genome of black pepper provides insight into piperine biosynthesis. Nat. Commun..

[24] Lamesch P., Berardini T.Z., Li D., Swarbreck D., Wilks C., Sasidharan R., Muller R., Dreher K., Alexander D.L., Garcia-Hernandez M. (2011). The *Arabidopsis* information resource (tair): Improved gene annotation and new tools. Nucleic Acids Res..

[25] Ghahramani Z. (2001). An introduction to hidden markov models and bayesian networks. In Hidden markov models. World Sci..

[26] Camacho C., Coulouris G., Avagyan V., Ma N., Papadopoulos J., Bealer K., Madden T.L. (2009). Blast+: Architecture and applications. BMC Bioinform..

[27] El-Gebali S., Mistry J., Bateman A., Eddy S.R., Luciani A., Potter S.C., Qureshi M., Richardson L.J., Salazar G.A., Smart A. (2018). The pfam protein families database in 2019. Nucleic Acids Res..

[28] Letunic I., Bork P. (2017). 20 years of the smart protein domain annotation resource. Nucleic Acids Res..

[29] Gasteiger E., Gattiker A., Hoogland C., Ivanyi I., Appel R.D., Bairoch A. (2003). Expasy: The proteomics server for in-depth protein knowledge and analysis. Nucleic Acids Res..

[30] Edgar R.C. (2004). MUSCLE: Multiple sequence alignment with high accuracy and high throughput. Nucleic Acids Res..

[31] Larkin M.A., Blackshields G., Brown N.P., Chenna R., McGettigan P.A., McWilliam H., Valentin F., Wallace I.M., Wallace I.M., Wilm A. (2007). Clustal W and clustal X version 2.0. Bioinformatics.

[32] Koichiro T., Glen S., Sudhir K. (2021). MEGA11: Molecular evolutionary genetics analysis Version 11. Mol. Biol. Evol..

[33] Nguyen L.T., Schmidt H.A., von Haeseler A., Minh B.Q. (2015). IQ-TREE: A fast and effective stochastic algorithm for estimating maximum-likelihood phylogenies. Mol. Biol. Evol..

[34] Subramanian B., Gao S., Lercher M.J., Hu S., Chen W.H. (2019). Evolview v3: A webserver for visualization, annotation, and management of phylogenetic trees. Nucleic Acids Res..

[35] Bailey T.L., Boden M., Buske F.A., Frith M., Grant C.E., Clementi L., Ren J., Li W.W., Noble W.S. (2009). MEME suite: Tools for motif discovery and searching. Nucleic Acids Res..

[36] Lee T.H., Tang H., Wang X., Paterson A.H., Paterson A.H. (2013). PGDD: A database of gene and genome duplication in plants. Nucleic Acids Res..

[37] Wang Y., Tang H., Debarry J.D., Tan X., Li J., Wang X., Lee T.H., Jin H., Marler B., Guo H. (2012). Mcscanx: A toolkit for detection and evolutionary analysis of gene synteny and collinearity. Nucleic Acids Res..

[38] Löytynoja A. (2021). Phylogeny-aware alignment with prank and pagan. Methods Mol. Biol..

[39] Wang D., Zhang Y., Zhang Z., Zhu J., Yu J. (2010). Kaks_calculator 2.0: A toolkit incorporating γ-series methods and sliding window strategies. Genom. Proteom. Bioinf..

[40] Griffiths-Jones S., Grocock R.J., van Dongen S., Bateman A., Enright A.J. (2006). miRBase: microRNA sequences, targets and gene nomenclature. Nucleic Acids Res..

[41] Zuker M. (2003). Mfold web server for nucleic acid folding and hybridization prediction. Nucleic Acids Res..

[42] Patanun O., Lertpanyasampatha M., Sojikul P., Viboonjun U., Narangajavana J. (2013). Computational identification of microRNAs and their targets in cassava (*Manihot esculenta* Crantz.). Mol. Biotechnol..

[43] Dai X., Zhuang Z., Zhao P.X. (2018). psRNATarget: A plant small RNA target analysis server (2017 release). Nucleic Acids Res..

[44] Langfelder P., Horvath S. (2008). WGCNA: An R package for weighted correlation network analysis. BMC Bioinform..

[45] Huang W., Sherman B., Lempicki R.A. (2009). Systematic and integrative analysis of large gene lists using DAVID bioinformatics resources. Nat. Protoc..

[46] Doncheva N.A.-O., Morris J.A.-O., Gorodkin J., Jensen L.A.-O.X. (2019). Cytoscape stringapp: Network analysis and visualization of proteomics data. J. Proteome Res..

[47] Meghwal M., Goswami T.K. (2013). *Piper nigrum* and piperine: An update. Phytother. Res..

[48] Smilkov K., Ackova D.G., Cvetkovski A., Ruskovska T., Vidovic B., Atalay M. (2019). Piperine: Old spice and new nutraceutical?. Curr. Pharm. Des..

[49] Panchy N.A.-O., Lehti-Shiu M.A.-O., Shiu S.A.-O.X. (2016). Evolution of gene duplication in plants. Plant Physiol..

[50] Ren R., Wang H., Guo C., Zhang N., Zeng L., Chen Y., Ma H., Qi J. (2018). Widespread whole genome duplications contribute to genome complexity and species diversity in angiosperms. Mol. Plant.

[51] Xing S., Quodt V., Chandler J., Höhmann S., Berndtgen R., Huijser P. (2013). SPL8 acts together with the brassinosteroid-signaling component bim1 in controlling *Arabidopsis thaliana* male fertility. Plants.

[52] Zhang Y., Schwarz S., Saedler H., Huijser P. (2007). SPL8, a local regulator in a subset of gibberellin-mediated developmental processes in *Arabidopsis*. Plant Mol. Biol..

[53] Zhang S.D., Ling L.Z., Yi T.S. (2015). Evolution and divergence of SBP-box genes in land plants. BMC Genom..

[54] Wang J.W., Czech B., Weigel D. (2009). miR156-regulated SPL transcription factors define an endogenous flowering pathway in *Arabidopsis thaliana*. Cell.

