# Supplementary material for: Genome-Wide Identification and Functional Exploration of SBP-Box Gene Family in Black Pepper (Piper nigrum L.)
Source: Genes (Basel). 2021 Oct 29;12(11):1740. doi: 10.3390/genes12111740 (PMC8625754; doi:10.3390/genes12111740)
Supplement: Supplementary file 1 [file genes-12-01740-s001.zip › Figure S1.pdf]

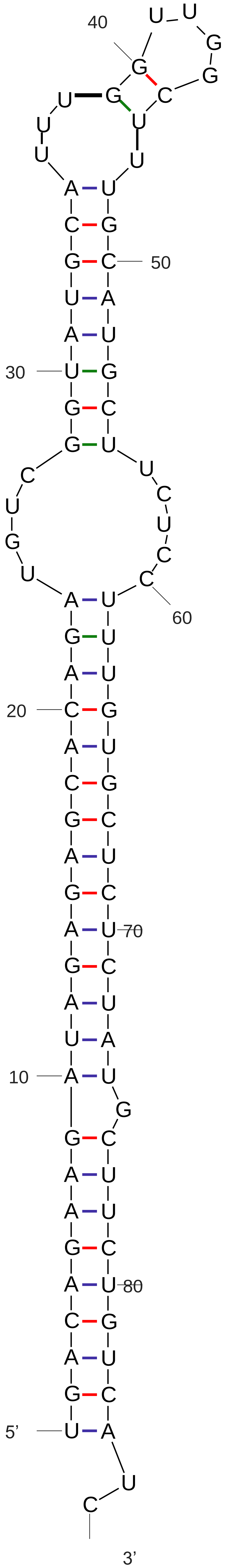

dG = -44.80 [Initially -44.80] Pn-miR156a

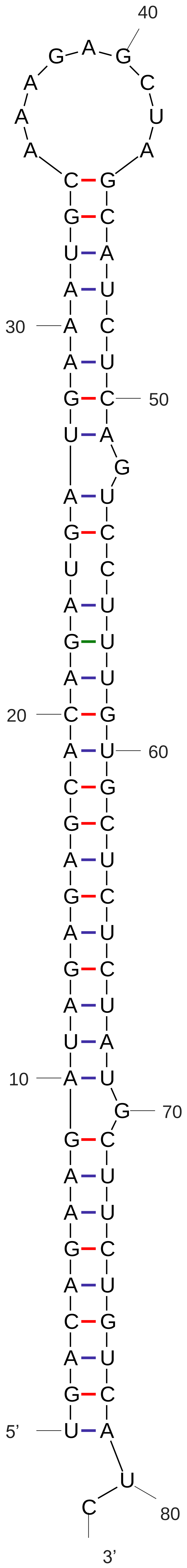

dG = -43.30 [Initially -43.30] Pn-miR156b

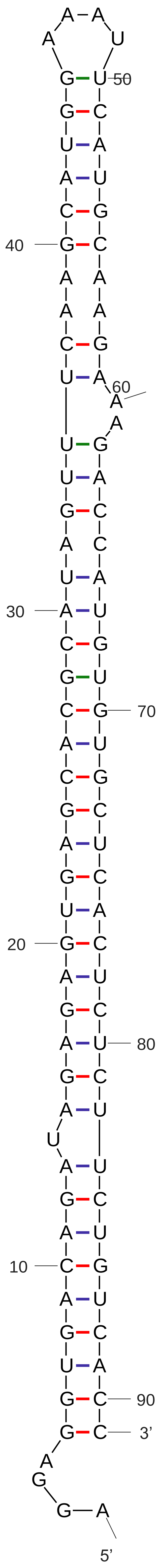

dG = -55.40 [Initially -55.40] Pn-miR156c

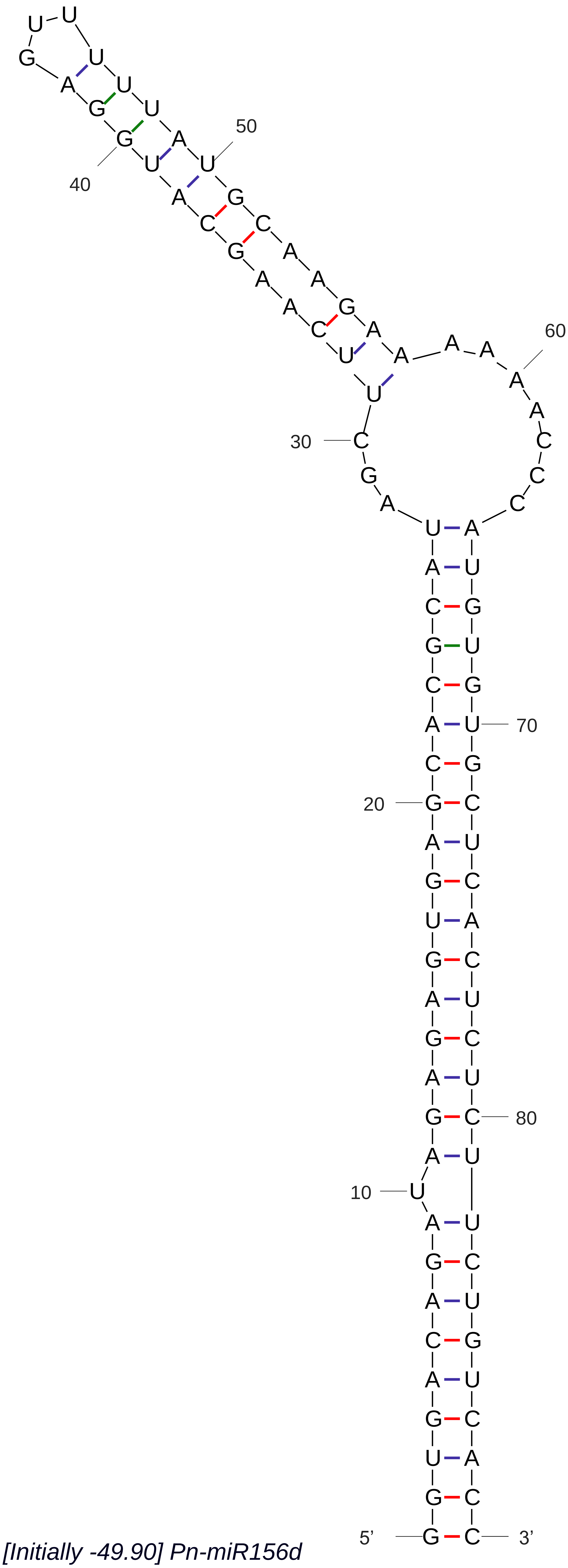

dG = -49.90 [Initially -49.90] Pn-miR156d

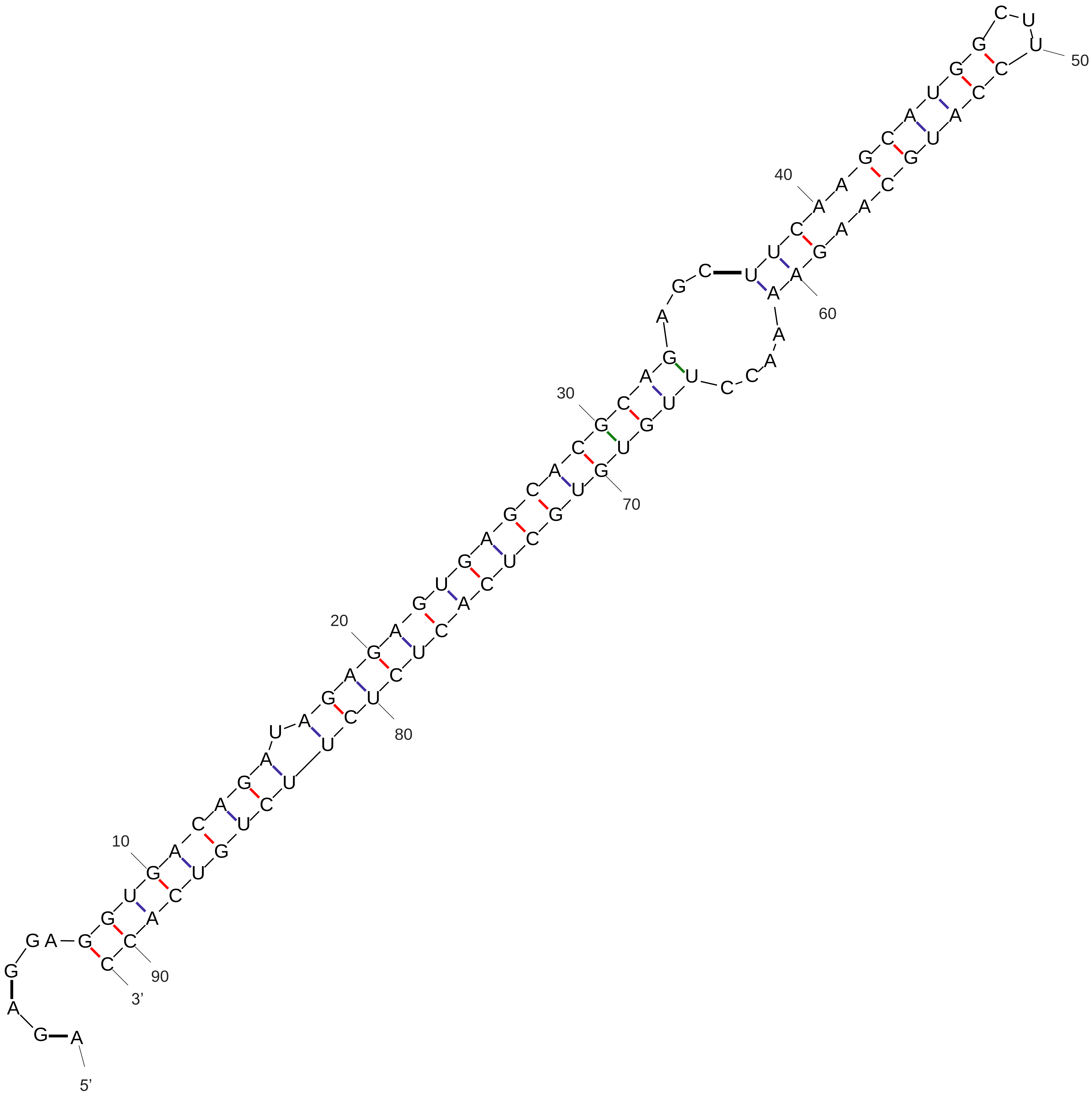

dG = -54.50 [Initially -54.50] Pn-miR156e

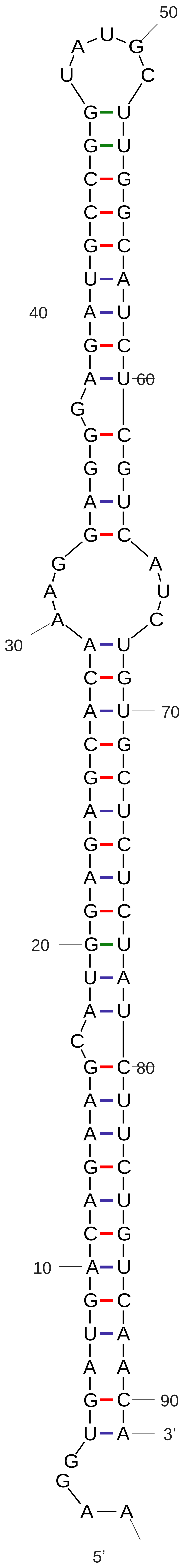

dG = -50.00 [Initially -50.00] Pn-miR156f

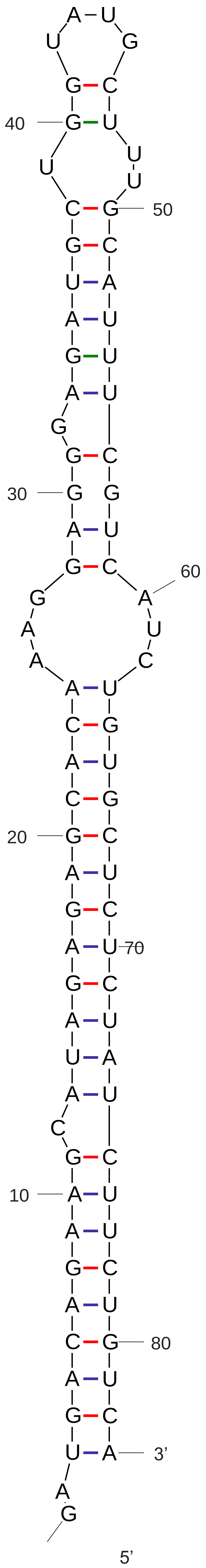

dG = -43.60 [Initially -43.60] Pn-miR156g

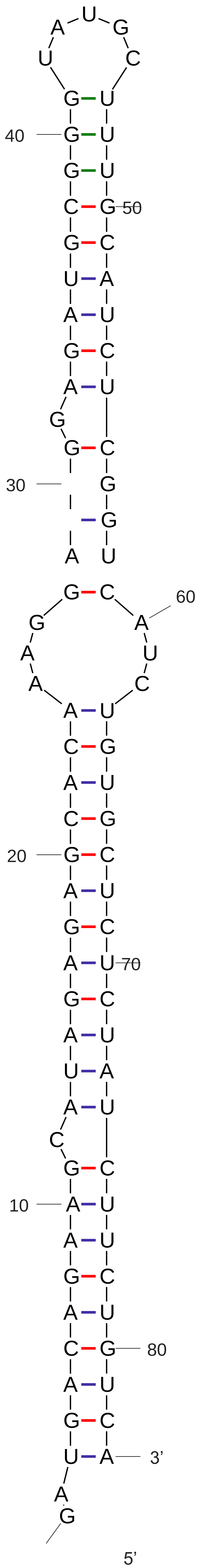

40
50
30
60
20
70
10
80
3'
5'

dG = -48.80 [Initially -48.80] Pn-miR156h

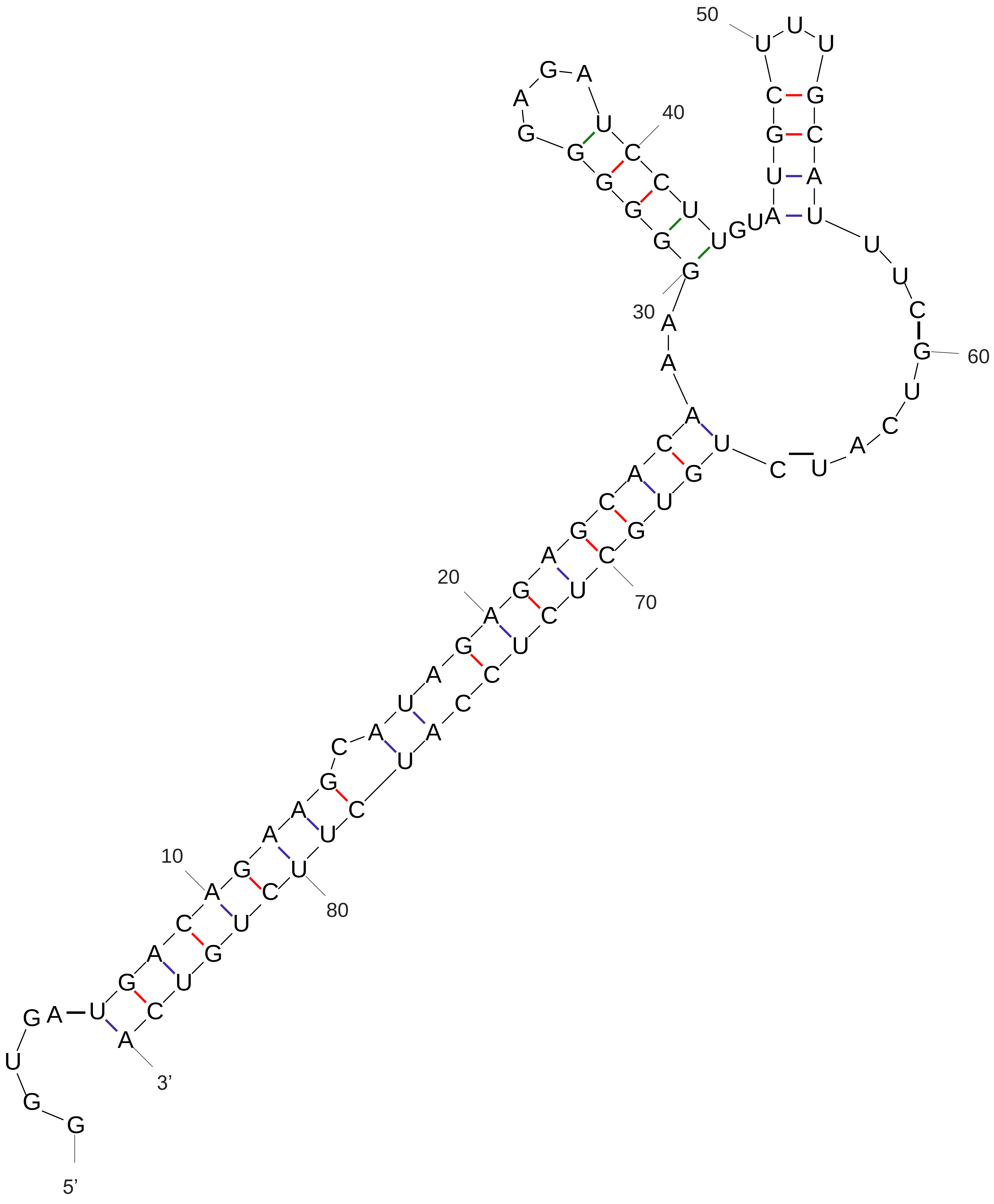

dG = -31.95 [Initially -35.40] Pn-miR156i

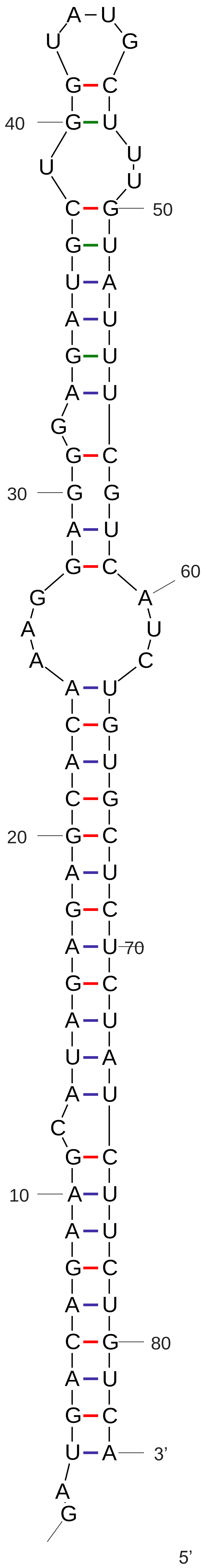

dG = -41.60 [Initially -41.60] Pn-miR156j

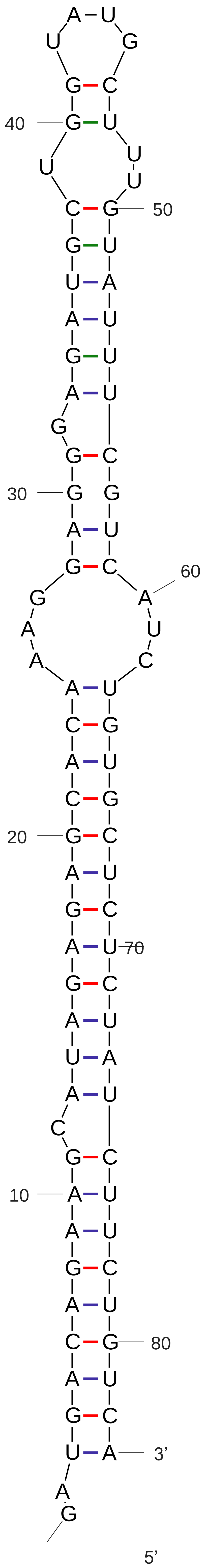

dG = -41.60 [Initially -41.60] Pn-miR156k

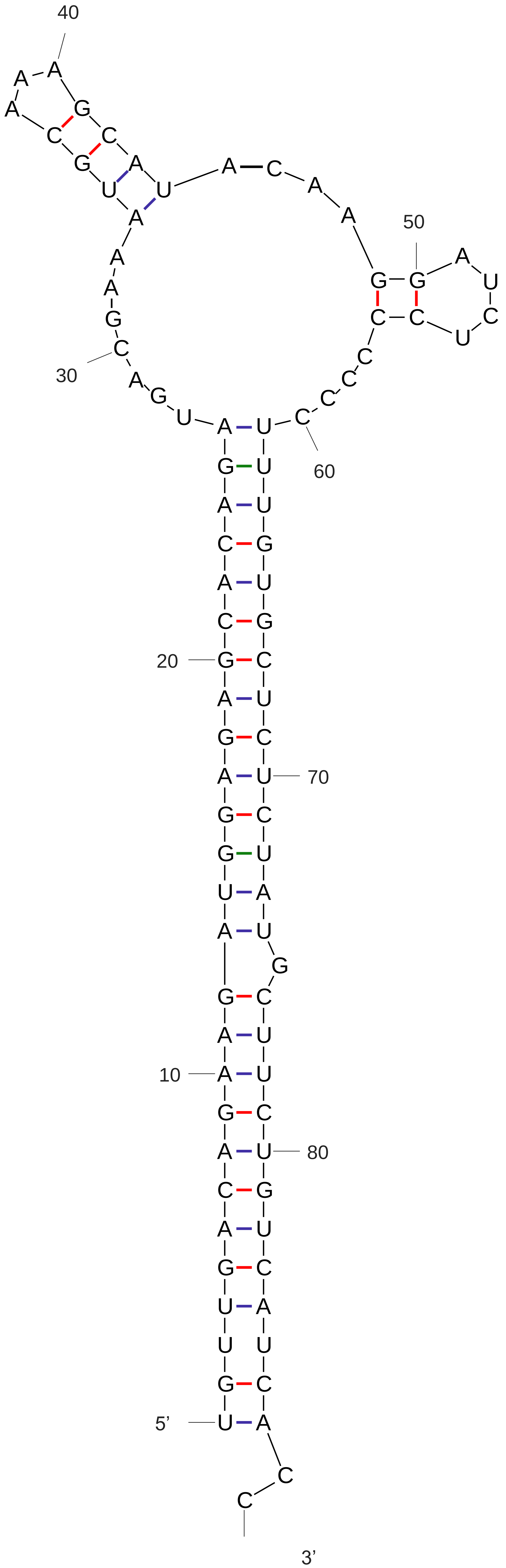

dG = -35.49 [Initially -38.70] Pn-miR156l

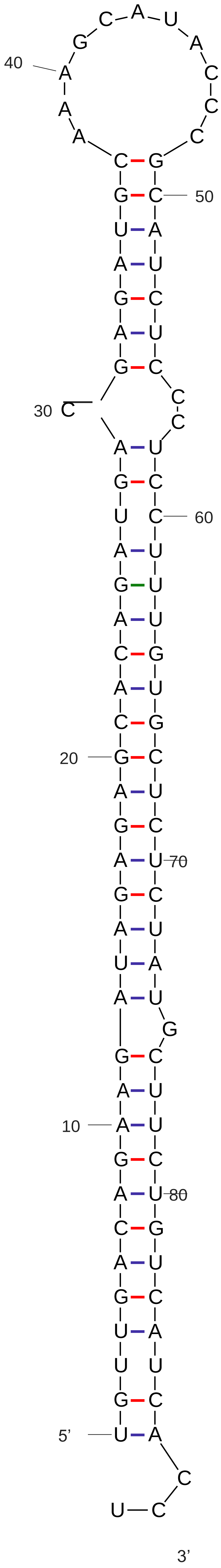

dG = -47.60 [Initially -47.60] Pn-miR156m

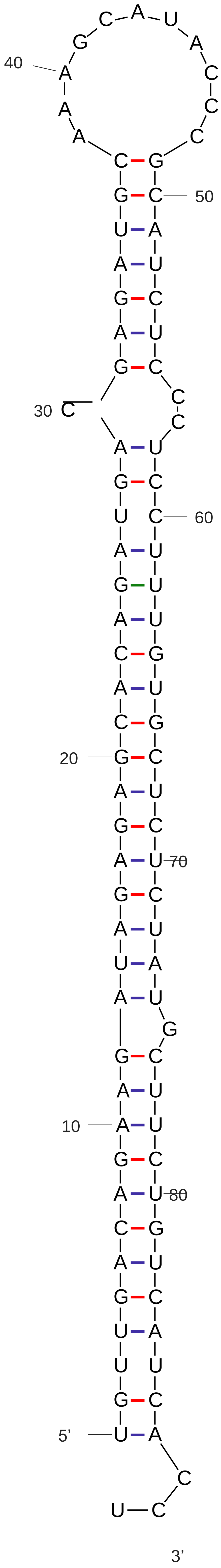

dG = -47.60 [Initially -47.60] Pn-miR156n

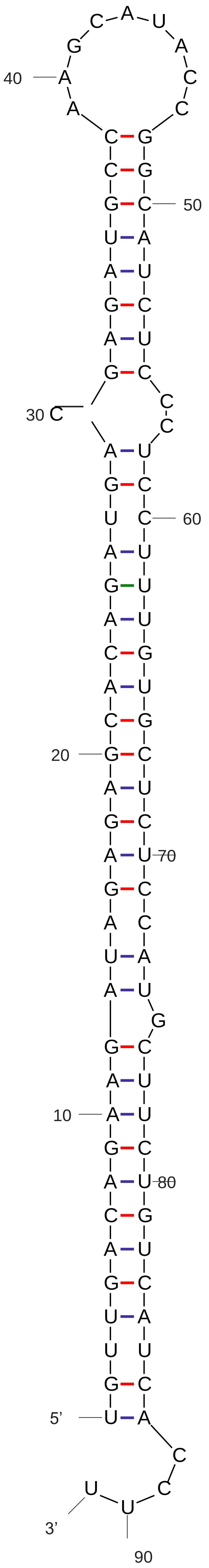

dG = -46.60 [Initially -46.60] Pn-miR156o

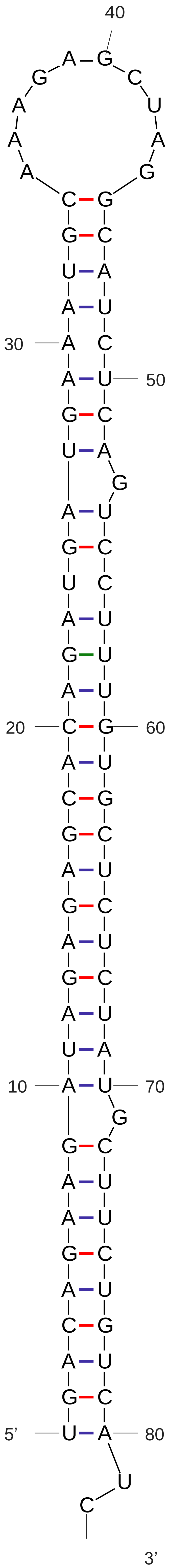

dG = -43.10 [Initially -43.10] Pn-miR156p

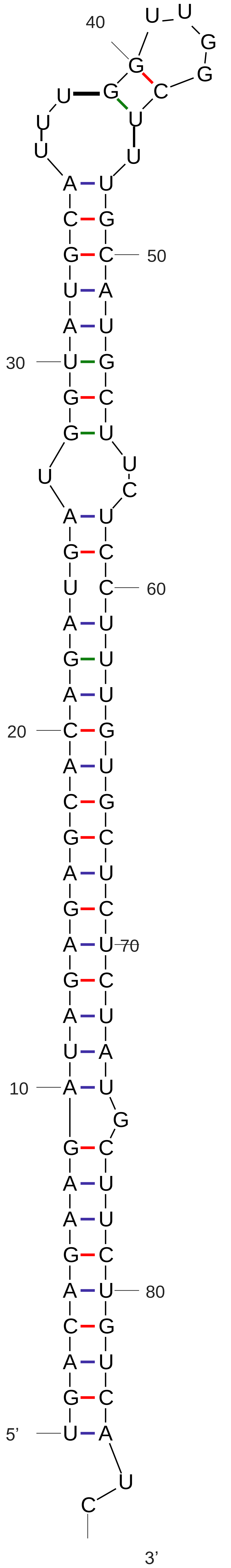

dG = -47.20 [Initially -47.20] Pn-miR156q

Figure S1. The prediction of RNA secondary structure of pepper miR156s.
