# Supplementary material for: Genome-Wide Identification and Functional Exploration of SBP-Box Gene Family in Black Pepper (Piper nigrum L.)
Source: Genes (Basel). 2021 Oct 29;12(11):1740. doi: 10.3390/genes12111740 (PMC8625754; doi:10.3390/genes12111740)
Supplement: Supplementary file 1 [file genes-12-01740-s001.zip › Table S1.pdf]

Table S1. The duplications of pepper *SBP* genes.

**Type**	**Gene1**	**Gene2**
**segment**	*PnSBP15*	*PnSBP16*
**segment**	*PnSBP26*	*PnSBP27*
**segment**	*PnSBP10*	*PnSBP11*
**segment**	*PnSBP3*	*PnSBP4*
**segment**	*PnSBP33*	*PnSBP34*
**segment**	*PnSBP24*	*PnSBP25*
**segment**	*PnSBP20*	*PnSBP21*
**segment**	*PnSBP30*	*PnSBP31*
**segment**	*PnSBP1*	*PnSBP4*
**segment**	*PnSBP20*	*PnSBP19*
**segment**	*PnSBP21*	*PnSBP19*
**segment**	*PnSBP28*	*PnSBP29*
**segment**	*PnSBP17*	*PnSBP18*
**segment**	*PnSBP17*	*PnSBP21*
**segment**	*PnSBP17*	*PnSBP20*
**segment**	*PnSBP17*	*PnSBP19*
**segment**	*PnSBP18*	*PnSBP21*
**segment**	*PnSBP18*	*PnSBP20*
**segment**	*PnSBP18*	*PnSBP19*
**segment**	*PnSBP8*	*PnSBP10*
**segment**	*PnSBP22*	*PnSBP23*
**segment**	*PnSBP13*	*PnSBP12*
**segment**	*PnSBP32*	*PnSBP34*
**segment**	*PnSBP12*	*PnSBP11*
**segment**	*PnSBP12*	*PnSBP10*
**segment**	*PnSBP5*	*PnSBP7*
**segment**	*PnSBP5*	*PnSBP6*
**segment**	*PnSBP6*	*PnSBP7*
**tandem**	*PnSBP24*	*PnSBP20*
**tandem**	*PnSBP25*	*PnSBP21*
