# Supplementary material for: Genome-Wide Identification and Functional Exploration of SBP-Box Gene Family in Black Pepper (Piper nigrum L.)
Source: Genes (Basel). 2021 Oct 29;12(11):1740. doi: 10.3390/genes12111740 (PMC8625754; doi:10.3390/genes12111740)
Supplement: Supplementary file 1 [file genes-12-01740-s001.zip › Table S2 miR.pdf]

Table S2. The information of predicted *miR156* genes in pepper genome.

ID	chromosome	start	end
Pn-miR156a	Pn11	27842833	27842918
Pn-miR156b	Pn15	24783644	24783724
Pn-miR156c	Pn19	17106468	17106378
Pn-miR156d	Pn19	17105920	17105831
Pn-miR156e	Pn19	17106765	17106675
Pn-miR156f	Pn23	1018520	1018430
Pn-miR156g	Pn23	1046854	1046772
Pn-miR156h	Pn23	1065414	1065332
Pn-miR156i	Pn23	1085641	1085556
Pn-miR156j	Pn23	1111206	1111124
Pn-miR156k	Pn23	3992571	3992489
Pn-miR156l	Pn23	4369605	4369693
Pn-miR156m	Pn23	4387815	4387904
Pn-miR156n	Pn23	4406378	4406467
Pn-miR156o	Pn23	4433017	4433107
Pn-miR156p	Pn66	190915	190996
Pn-miR156q	Pn9	9331714	9331799
