# Supplementary material for: Genome-Wide Identification and Functional Exploration of SBP-Box Gene Family in Black Pepper (Piper nigrum L.)
Source: Genes (Basel). 2021 Oct 29;12(11):1740. doi: 10.3390/genes12111740 (PMC8625754; doi:10.3390/genes12111740)
Supplement: Supplementary file 1 [file genes-12-01740-s001.zip › Table S3 target.pdf]

Table S3. The distribution of miR156 binding sites in pepper *SBP* genes.

Target	binding sites	Target start	Target end
PnSBP2	GUGCUCGCUCUCUUCUGUCA	887	906
PnSBP12	GUGCUCUCUCUCUUCUGUCA	797	816
PnSBP15	GUGCUCUCUCUCUUCUGUCA	626	645
PnSBP21	GUGCUCUCUCUCUUCUGUCA	635	654
PnSBP20	GUGCUCUCUCUCUUCUGUCA	635	654
PnSBP8	GUGCUCUCUCUCUUCUGUCA	773	792
PnSBP13	GUGCUCUCUCUCUUCUGUCA	761	780
PnSBP4	GUGCUCUCUCUCUUCUGUCA	746	765
PnSBP17	GUGCUCUCUCUCUUCUGUCA	683	702
PnSBP11	GUGCUCUCUCUCUUCUGUCA	779	798
PnSBP14	GUGCUCUCUCUCUUCUGUCA	797	816
PnSBP1	GUGCUCUCUCUCUUCUGUCA	815	834
PnSBP6	GUGCUCUCUCUCUUCUGUCA	980	999
PnSBP10	GUGCUCUCUCUCUUCUGUCA	2450	2469
PnSBP18	GUGCUUUCUCUCUUCUGUCA	764	783
PnSBP16	GUGCUCUCUCUCUCCUGUCA	479	498
PnSBP3	GUGCUCCCCCUCUUCUGUCA	620	639
